# Pain-Related Fear: A Critical Review of the Related Measures

**DOI:** 10.1155/2011/494196

**Published:** 2011-11-15

**Authors:** M. Lundberg, A. Grimby-Ekman, J. Verbunt, M. J. Simmonds

**Affiliations:** ^1^Division of Occupational Orthopedics, Department of Orthopedics, Sahlgrenska University Hospital, University of Gothenburg, Göteborg, Sweden; ^2^Occupational and Environmental Medicine, Sahlgrenska University Hospital and Academy at University of Gothenburg, Göteborg, Sweden; ^3^Department of Rehabilitation Medicine, Maastricht University Medical Center and Research School CAPHRI, M D Maastricht University, 6211 LK Maastricht, The Netherlands; ^4^A.T. Still Research Institute, A.T. Still University, Kirksville, MO 63501, USA; ^5^Department of Physical Therapy, University of Texas Health Sciences Center, San Antonio, 7703 Floyd Curl Drive San Antonio, TX 78229-3900, USA Department of Physical Therapy, University of Texas Health Sciences Center, San Antonio, 7703 Floyd Curl Drive San Antonio, TX 78229-3900, USA

## Abstract

*Objectives*: In regards to pain-related fear, this study aimed to: (1) identify existing measures and review their measurement properties, and (2) identify the optimum measure for specific constructs of fear-avoidance, pain-related fear, fear of movement, and kinesiophobia. *Design*: Systematic literature search for instruments designed to measure fear of pain in patients with persistent musculoskeletal pain. Psychometric properties were evaluated by adjusted Wind criteria. *Results*: Five questionnaires *(Fear-Avoidance Beliefs Questionnaire (FABQ), Fear-Avoidance of Pain Scale (FAPS), Fear of Pain Questionnaire (FPQ), Pain and Anxiety Symptoms Scale (PASS), and the Tampa Scale for Kinesiophobia (TSK))* were included in the review. The main findings were that for most questionnaires, there was no underlying conceptual model to support the questionnaire's construct. Psychometric properties were evaluated by diverse methods, which complicated comparisons of different versions of the same questionnaires. Construct validity and responsiveness was generally not supported and/or untested. *Conclusion*: The weak construct validity implies that no measure can currently identify who is fearful. The lack of evidence for responsiveness restricts the current use of the instruments to identify clinically relevant change from treatment. Finally, more theoretically driven research is needed to support the construct and thus the measurement of pain-related fear.

## 1. Introduction

Over the last decennia, fear of pain and/or injury has become an integral part of our understanding in the explanation of disability in patients with persistent musculoskeletal pain [1]. Pain-related fear has been identified in the general population [2] as well as in various patients' groups with persistent musculoskeletal pain [3–9]. Subsequently, treatment strategies have been developed to reduce fear and disability in patients with low back pain [10, 11], as well as neuropathic pain [4]. In the clinical setting fear is acknowledged as an important aspect in patients' disability which needs to be addressed to achieve successful outcome. Although the role of fear in pain-related disability seems thus well established, consensus concerning proper assessment and interpretation of fear in relation to pain is currently still lacking.

One of the reasons for this is the fact that fear in relation to pain has been described with a variety of conceptual definitions among which pain-related fear, fear-avoidance beliefs, fear of movement, and kinesiophobia are the most commonly used. These are, however, constructs rather than a disorder or other pathological state in and of itself. The relationship between fear and pain was first described by Lethem et al. 1982 in *“the fear-avoidance model of exaggerated pain perception”* [12], in which fear and pain were both presented as associated with behaviour through avoidance learning. Almost a decade later, Kori et al. presented their thoughts about *kinesiophobia* [13]. Kinesiophobia was originally defined as a condition in which a patient has* “an excessive, irrational, and debilitating fear of physical movement and activity resulting from a feeling of vulnerability to painful injury or reinjury”*. The model that has gained most interest among researchers and clinicians alike is the “*cognitive-behavioural fear-avoidance model”* [14]. In that model *fear of movement* was identified as an important factor to result in disability, disuse, and depression in patients with persistent musculoskeletal pain. A subsequent construct is *pain-related fear* [15], defined as fear that *incorporates fear of pain, fear of injury, fear of physical activity, and so forth*. Closely related constructs are “fear-avoidance beliefs” [16] and “pain-related fear-avoidance beliefs” [17], but for none of them a conceptual definition is available. In the literature, the above-mentioned constructs are often used interchangeably, although they are not synonyms. Therefore, it seems that, currently, the conceptual framework of the various constructs used in relation to fear and pain are far from clear.

Despite the limited evidence available concerning the underlying constructs, over years several questionnaires have already been developed for the assessment of fear in relation to pain [16, 18, 19]. The Tampa Scale for Kinesiophobia (TSK) is the oldest existing measure and was designed 1991 by Miller et al. [19] to measure the patient's subjective estimation of kinesiophobia. The *Fear-Avoidance Beliefs Questionnaires (FABQ)* [16] and the *Pain Anxiety Symptoms Scale (PASS)* [18] were subsequently developed. These questionnaires are currently all used in research as well in clinical practice, in order to identify problem areas and to aid in the design of targeted treatment strategies for the patient. However, no systematic evaluation of the psychometric properties of the existing measures has ever been performed. 

Therefore, the aims of the present study were threefold; first to identify the existing measures, second to review the measurement properties, and thirdly to identify the optimum measure for specific constructs fear-avoidance, pain-related fear, fear of movement and kinesiophobia.

## 2. Methods

### 2.1. Search Strategy

A systematic literature search for instruments specifically designed to measure fear of pain in patients with persistent pain was performed. The databases used for this search included PubMed, Cinahl, Embase, PsycINFO, and articles published between January 1990 to June 2009 were saved. The search was conducted in two steps. First the search items *“assessment”* and *“pain”* were combined with the subordinate terms *“fear avoidance”, “pain-related fear”, “fear of movement”*, and *“kinesiophobia”.* Once the relevant questionnaires were found, the second step was performed to assess the measurement properties of the various questionnaires using the search terms *“reliability”, “validity”,* and *“psychometric”*.

### 2.2. Inclusion Criteria

Article abstracts were read to determine which questionnaires were included. An article was included if it contained research on adults (>18 years of age) with persistent pain and if the article was written in the English language. All articles that met inclusion criteria were reviewed by the two authors (M. Lundberg and A. Grimby-Ekman.). In case of disagreement between the reviewers, an independent reviewer (J. Verbunt) was consulted in order to obtain a consensus.

### 2.3. Assessment Criteria

The review criteria included an evaluation of the following psychometric steps: *Conceptual and measurement model, reliability, validity, responsiveness, interpretability, practicality, and cross-cultural applicability *[20]. To assess the levels of the psychometric steps mentioned above we used criteria adjusted after Wind et al. [21] (Table 1). 

### 2.4. Psychometric Terms Defined for This Study

The following definitions were used in the current study. 

A *conceptual model* is a rationale for and a description of a concept(s) that the measure is intended to assess and describes the relationship between the concepts included [20].

*Reliability* is the extent to which a measurement is consistent and free from error [22, 23]. Reliability can be assessed based on various subconstructs. For the purpose of this study we have chosen to divide reliability into *internal consistency* [24] and *reproducibility* [20]. 

*Measurement Validity *refers to the extent to which an instrument measures what it is intended to measure [22, 23, 25]. Validity can be assessed based on different components [22, 23, 26]. For the purpose of this study validity will be presented as *face-, content-, construct-, and criterion-related validity* [20]. 

*Responsiveness describes *the instrument's ability to detect change. Less frequently used, but clinically important terms are *interpretability *and* practicality. *Interpretability describes the degree to which one can assign easily understood meaning to an instrument's quantitative scores. Practicality refers to aspects as time, effort, and other demands placed on those on which the instrument is being administered (respondent burden) or on those who administer the instrument (administrative burden). 

The *cross-cultural adaptation* is about assessment of conceptual and linguistic equivalence and evaluation of their psychometric properties. For the purpose of this study, we have chosen to evaluate the cross-cultural adaptation separately for each instrument.

## 3. Results

The initial search strategy identified 588 abstracts from the PubMed, Cinahl, Embase, PsycINFO, and Web of Science databases. After removing duplicates, 37 abstracts were retrieved for further review. Of the 37 articles, 13 articles addressed the FABQ, one article the FAPS, 2 articles the FPQ, 10 articles the PASS, and 12 articles the TSK. The results are presented as a brief summary of the assessment of the measurement properties of all questionnaires (Table 2). In addition, a more detailed overview of the included articles is presented in Table 3. 

### 3.1. Questionnaires Identified in Relation to the Conceptual Definitions

**Figure 1:**
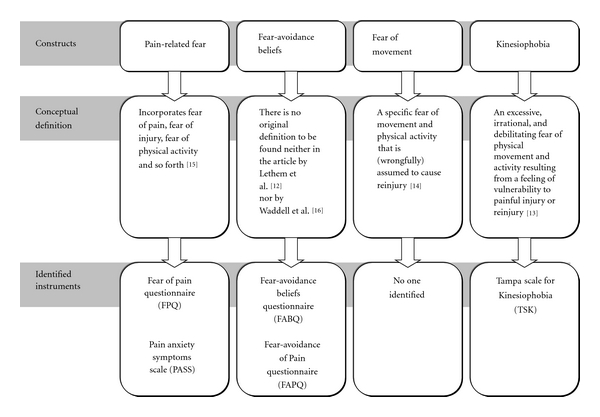
A schematic overview of the relationship between the constructs “fear-avoidance beliefs”, “pain-related fear”, “fear of movement,” and “kinesiophobia” and the identified instruments.

*Fear of Pain Questionnaire (FPQ) *and *Pain Anxiety Symptoms Scale (PASS) *were identified as being designed to measure the construct “pain-related fear”. The* Fear Avoidance Beliefs Questionnaire (FABQ) *and* the Fear-Avoidance of Pain Scale (FAPS)* were identified as designed to measure the construct “fear-avoidance beliefs”. The *Tampa Scale for Kinesiophobia (TSK) *was identified to measure “kinesiophobia”. No questionnaire was found to assess the construct “fear of movement”.  Figure 1 demonstrates the relationship between the constructs and the identified measures. 

### 3.2. Descriptions of the Included Questionnaires

The *Fear-Avoidance Beliefs Questionnaire* (FABQ) was originally developed by Waddell et al. [33] to assess fear-avoidance beliefs in patients with back pain. The original version of the FABQ contains 16 items divided into two subscales: fear-avoidance beliefs about physical activity (5 items) and fear-avoidance beliefs about work (11 items). Each of the 16 items are rated on a seven-point Likert Scale (“do not agree at all” = 0 to “completely agree” = 6). The FABQ is available in nine languages, with each language version evaluated in relation to its psychometric properties.

The *Fear Avoidance of Pain Scale (FAPS) *was constructed by Crowley and Kendall [42] to measure fear avoidance of activities. The FAPS comprises 21 items ranging from 0 (never) to 6 (all the time). Patients are asked to rate how often the mentioned activity occurs. 

The *Fear of Pain Questionnaire (FPQ)* is presented to measure fear of pain and is based on the work of Lethem et al. [12]. The FPQ versions used in the two included articles consist both of a total number of 30 items, but the individual items included differ however in both articles. The FPQ items are statements briefly describing painful situations, and the patient is asked to mark the “amount of fear” on a scale of 1 (not at all) to 5 (extreme) for each item. Three subscales are reported: fear of minor, severe, and medical pain. A total score is used. 

The *Pain Anxiety Symptoms Scale (PASS)* was originally designed to assess fear of pain [18] in relation to the three response modalities: cognitive, physiological, or motor response domains. The patients are asked to rate their score for each item on a scale from 0 (never) to 5 (always). Items on the PASS are measured on a 6-point Likert scale. Different versions of the PASS exist including the 53-item questionnaire and an abbreviated version consisting of 20 items.

The *Tampa Scale of Kinesiophobia (TSK)* was designed to measure kinesiophobia [19]. The original TSK consists of 17 items. Each item is evaluated on a 4-point Likert scale with scoring alternatives ranging from “strongly disagree” to “strongly agree”. A total sum is calculated after inversion of the individual scores of items 4, 8, 12, and 16. The total score can vary from 17 to 68. The TSK is available in four languages, but can contain various versions of the TSK. For instance, the various English language versions contain 4, 11, 13, or 17 items.

### 3.3. General Summary according to the Psychometric Properties for All Questionnaires

None of the articles reviewed provided a rationale reasoning regarding the conceptual and measurement model related to the questionnaire. Extensive work on the definition of psychometric properties has been performed on the FABQ and the TSK. At present, the FABQ appeared to be the best available measure, in terms of psychometric properties, to measure the concept “fear-avoidance beliefs”, the PASS seems to be the best available measure to measure “pain-related fear”, and the TSK is the best available to measure “kinesiophobia”. As shown in Table 2, for various questionnaires information concerning reliability and validity is often lacking. In addition, for almost all measures evidence to confirm its responsiveness is lacking. Measures of interpretability and practicality were rarely or never addressed and hence not evaluated. Each identified measure is analysed in depth below.

### 3.4. Fear-Avoidance Beliefs Questionnaire (FABQ)

In total, 16 articles were included in the review of the FABQ. These articles addressed the psychometric properties of the Chinese [29, 30], Dutch [31, 32], English [16, 34], French [35], German [36–38], Greek [39], Hebrews [40], Norwegian [27], and Spanish language versions [41], with each language version presented separately (Tables 2 and 3). 

As shown in Table 2 the internal consistency of the FABQ_total_ as well as its subscale FABQ_work_ was assessed as high in all language versions. However, the internal consistency of the subscale FABQ_physical activity_ appeared to be only low to moderate in most language versions. The FABQ reproducibility as reported ranged from low to high, whereas the reported FABQ validity could be classified as low. The quality of the FABQ validity analysis procedures as performed could be classified as compromised. Study objectives were often not clearly focussed, which complicated identifying the validation procedure as performed. As shown in Table 2, only limited information appeared to be available related to the FABQ's content validity. Most often, the construct validity was assessed using factor analyses. Various factor solutions were presented ranging from one to four, with two being the most common factor solution. The items within the factor solutions varied, but were mostly robust. As shown in Table 3, a variety of measures were used to either converge or diverge in relation to the FABQ. Testing conditions were not reported in any of the studies. 

The criterion-related validity, both concurrent and predictive, of the FABQ was ranging from low to moderate. The presentation of the rationale for the choice of the criterion-related measures was often lacking, which complicated the interpretation. 

### 3.5. Fear Avoidance of Pain Scale (FAPS)

One article appeared to be available that evaluated the psychometric properties of the FAPS. Based on this information, the internal consistency is considered as high whereas its reproducibility seems low. Concurrent validity was ranging from low to moderate. Moreover, the evidence supporting validity of the FAPS is poor in all versions. It is also unclear what subscale of the FAPS would be preferable to use.

### 3.6. Fear of Pain Questionnaire (FPQ)

Our selection of articles identified 16 articles that evaluated psychometric properties of the FPQ. However, only two of these articles [43] fulfilled our inclusion criteria. The FPQ measures fear of pain and is to be based on the work of Lethem et al. [12]. However, a remarkable finding seems to be the fact that several of the selected articles refer to one specific document, classified as proceedings of a meeting, in which the origin of the FPQ [45] is reported to be described. The availability of this FPQ key manuscript seemed however rather limited since we were not able retrieve it, even after a thorough search in the international literature. The FPQ as referred to in the two articles included in the final selection consisted of 30 items. All items describe painful situations, and the patient is asked to mark the “amount of fear” he/she is experiencing related to each item on a scale of 1 (not at all) to 5 (extreme). The three subscales mentioned are fear of minor, severe, and medical pain. A total score is used. As reported the internal consistency varied between low in the medical scale to high in the total scale. The content validity was considered low. 

### 3.7. Pain Anxiety Symptoms Scale (PASS)

In total, 12 articles were included in the review of the PASS. Articles discussing the psychometric properties of the English [18, 28, 34, 47–49] and Dutch language [46] versions were addressed (Table 1). The reliability was high in all PASS language versions, except for the English 20-item version of the subscale PASS_physiological symptoms of anxiety _which was reported to be only moderate. As for the FABQ, the description of the validation procedure for the PASS was often lacking in the articles reviewed. In addition, the diversity of versions of the PASS complicated comparisons between studies. Content validity ranged between low and moderate in the English and the French versions of the PASS. Construct validity was found to be low in the combined Dutch and American study [46]. As shown in Table 3, a variety of measures were used as a criterion variable in the various validation procedures. Again motivation for the selection of these criterion measures was often not available. Concurrent validity was low to moderate in the Dutch and English version of the PASS [59]. Responsiveness was addressed in only one study. In addition to the original version, two versions of the PASS are available in Dutch.

### 3.8. Tampa Scale for Kinesiophobia (TSK)

A total number of 12 articles addressed the psychometric properties of the Dutch [50–52], English [53–56], Norwegian [57], and Swedish version of the TSK [7]. Reliability was not evaluated at all in the Norwegian version of the TSK. The reliability of the English version ranged from low in the 13-item version subscale TSK_tsf_ [55] to high in TSK_total. _The fact that there are three different variations of the English language version available complicates its final qualification. The reliability of the various Dutch versions ranged from low on the 13-items subscale TSK_pathologic somatic focus _to high on the TSK_total_ in a group of patients with Fibromyalgia. The support for reliability of the Swedish version was found to be high in a group of patients with persistent low back pain [7]. Validity was found to be low in all versions of the TSK. 

## 4. Discussion

Five questionnaires, *Fear of Pain Questionnaire (FPQ), Pain Anxiety Symptoms Scale (PASS)*, *Fear-Avoidance Beliefs Questionnaire (FABQ), *the* Fear Avoidance of Pain Scale (FAPS),* and the *Tampa Scale for Kinesiophobia (TSK), *were identified to assess fear in relation to pain. All questionnaires had weaknesses in relation to their psychometric properties, poor reliability and validity indicating a lack of construct validity. At present the FABQ seems to be the best available questionnaire to measure the concept “fear-avoidance beliefs”, the PASS seems to be the best available questionnaire to measure “pain-related fear”, and the TSK is the best used to measure “kinesiophobia”. 

### 4.1. Conceptual and Measurement Models

As shown in this study the description of the underlying conceptual model appeared to be poor (FABQ, FAPQ, PASS) to nonexistent (TSK). The fact that no or only limited information is available concerning the description of the conceptual model has direct impact on the manner in which the concepts are consecutively operationalised and measured. As a consequence of that, it is currently unclear how individual scores obtained from either of these questionnaires could be interpreted for research and clinical settings. Although in clinical practice several of the presented questionnaires are already frequently in use in order to screen individual patients for fear related to pain, based on the results of this review, it could be concluded that the interpretation of the scoring has to be performed with caution. For example, what would be the clinical relevance of a high score on the FABQ? Based on the results of the current review, it can only be concluded that in such a situation, a patient who has elevated scores on the FABQ cannot be interpreted as having high fear-avoidance beliefs. A reported observation of Linton et al. [60] agrees with this finding. In one of their studies, patients showed a clinical relevant decrease in their fear-avoidance beliefs as observed by the rehabilitation staff, although this change was not represented in a change in TSKscoring. Patients continued to have high scores even after successful participation in the treatment. This finding may suggest that the construct validity of the TSK version used was poor as the questionnaire did not target fear as it was intended to or that the measure was not sensitive enough to pick up the actual clinical change. It can be concluded that there is an ambiguity in relation to the conceptual framework, and there are several different constructs to define what fear is in relation to pain.

It should be emphasized that none of the measures seem to be explicitly based on the frequently referred to and well-established “*cognitive-behavioural fear-avoidance model”* [14]. All of the identified questionnaires (FABQ, FAPS, FPQ, PASS and TSK) were already designed before the introduction of Vlaeyen's fear avoidance model in 1995. This fear avoidance model has been further elaborated on by several other researchers [61–63]. In addition, two recent models have tried to incorporate alternative activity-related behavioural strategies, among these are the Ergomania model [64] and the Avoidance-Endurance model [65]. In summary, there seems to be a discrepancy between the moment the measures and the conceptual models available on fear related to pain were introduced. This leads to the question, what the questionnaires in their current versions really measure. 

### 4.2. The Link between Models and Measures

At this stage, the reader might wonder whether the questionnaires can be used at all. Closely linked to the conceptual framework is the validity of each questionnaire. In the current study, the evaluation of the evidence concerning validity was complex. It must however be noted that establishing validity of an instrument is extremely difficult [26] what seems to imply that no questionnaire can be labelled as 100% valid [66]. In the current study, firstly, the validation procedure was often poorly described, complicating quality judgment of this procedure. Some of the authors, however, succeeded to guide the reader through the procedure [27, 54]. Secondly, a variety of measures were used as external criterion in the concurrent/criterion validity process. As shown in Table 3 all of the selected questionnaires were validated against other questionnaires as part of the validation process. However, the validity of the instrument used as the “gold standard” appeared to be in many occasions less established as it was assumed. Based on the results of the current review, it was shown that the FABQ, PASS, and TSK were all used to evaluate the validity of a reference instrument which has been included during the original validity evaluation of the questionnaire itself. Based on this circular reasoning, it can however only be concluded that both instruments measure the same construct, but whether this construct is indeed the desired construct is still unknown. For instance, studying the association between the TSK and the FABQ will result in information regarding the construct validity [67] of the questionnaires, but will not evaluate the concurrent/criterion validity for which a gold standard scale is required. In the articles reviewed, most authors referred indeed to the validation as an evaluation of construct/convergent rather than concurrent/criterion validity. However, it should be noted that even if the term for the validation is used properly, the weakness of construct/convergent validity is still present. Moreover, whether a test is valid or not is ultimately a matter of opinion based on the evidence available describing its validity [26].

Some researchers have argued that an evaluation of construct validity is the only proper way to evaluate the validity of an instrument [22, 26]. During construct validity evaluations, three essential steps have to be taken. Firstly, the domain of observables related to the construct has to be specified; secondly, the extent to which observables tend to measure the same construct has to be determined; thirdly studies and/or experiments have to be performed to determine the extent in which supposed measures of the constructs are consistent with “best guesses” about the construct. According to Nunnally and Bernstein [22], researchers often tend to develop a measure of a construct and then leap to the third aspect, for example, correlating a particular measure of anxiety with a particular measure of shyness instead of tightly defining the initial domain of observables for the construct. The challenge that lies ahead is to pool all the observations together by all these measures and place them into a new elaborated framework. 

### 4.3. Responsiveness

Based on the results of our review, it can be concluded that the information currently available on psychometric properties of the instruments is too limited to establish their quality as diagnostic tests. For example, at the moment evidence-based cut-off scores for the various instruments are still not available. In addition, the limited information regarding responsiveness of the questionnaires complicates the use in clinical practice, since information on a clinically relevant change of an instrument is not available. Information on responsiveness was only available for the FABQ (German, French, Norwegian, Spanish, and Chinese versions and the TSK 11-item English version). Whether these instruments are applicable for screening patients for pain-related fear seems an important topic for future research. To support the appropriateness as an outcome measure, more information must be gathered on the responsiveness of the various measures.

### 4.4. Interpretability and Practicality

Both interpretability and practicality are seldom discussed in published articles, even though the evaluation of both constructs seem highly relevant in the questionnaire selection process. In the present review, these issues were only addressed in relation to the Spanish version of the FABQ [41]. In clinical practice, both criteria seem relevant and need to be highlighted for future research.

### 4.5. Cross-Cultural Applicability

During cross-validation, both language and cultural differences should be taken into account. For example, can a questionnaire which has been reported as reliable and valid in Chinese based on cross-cultural validation between languages be used throughout entire China, representing various intercultural differences within this enormous country? As for the TSK, in Sweden and Norway, two countries with a comparable cultural background, two separate versions of the TSK are used, whereas in Belgium and Holland, the same language versions are used, without considering possible cultural differences. Furthermore, the focus on language excludes immigrants, and there is a limitation in the generalization of the results.

### 4.6. Methodological Considerations

This review was based on an evaluation of psychometric properties. It is evident that there is no such thing as a “gold standard” criteria for psychometric testing. Even within the domain of research on psychometric evaluation, there is no consensus as to what should be included in an analysis of reliability and validity [66]. We therefore chose to base our analyses of the psychometric properties on a modified version of the Wind-criteria [21]. For this purpose, the original Wind criteria was adjusted based on the modification used in criteria of Grotle et al. [68] and Larsen and Marx [69]. We would also like to clarify that the rationale for using criteria instead of simply presenting the data as shown in Table 3 was to provide a guidance for the reader who is trying to decide which measure to use.

There are, however, other methods available to evaluate reliability and validity. Psychometric theory assumes that the data is normally distributed and treated as data on at least an interval level, while others argue that data from questionnaires are ordinal data [70, 71]. Svensson [72] has developed a family of non-parametric rank-invariant methods that are valid for all types of ordered data without assumptions about their distribution. Bunketorp et al. [58] applied Svensson's method to evaluate the reliability of a slightly different version of the TSK-SV and found it reliable.

Another model available for evaluation of measurement quality is the Rasch Model. [73], Rasch models [50, 52] are logistic models in item response theory in which a person's level on a latent trait and the various items on the same latent variable can be estimated independently. The Rasch model was applied to the Norwegian version of the TSK by Damsgard et al. [57].

### 4.7. Limitations of the Study

Other instruments often presented in association with the evaluated constructs are the *Photographs Series of Daily Activities* (PHODA) [74] the *Pain Beliefs Screening Instrument* (PBS) [75], *Örebro Musculoskeletal Pain Questionnaire* [76], and the *Pain and Impairment Relationship Scale *(PAIRS) [77]. These instruments were not included in this review for various reasons. The PHODA was not identified as part of the search, since it was originally presented in Dutch. Furthermore, it is designed to determine the level of perceived harmfulness of various physical activities and movements. Both the PBS and the Örebro Musculoskeletal Pain Questionnaire are designed to screen patients at risk of developing persistent pain whereas the PAIRS was designed to assess beliefs about chronic pain and functional impairment and were not included for further evaluation in the current review. 

Another issue to be raised is the statement that there is an (over)reliance on self-reports [78] for both constructs (fear and pain). Verbal reports as well as the other methods (observation by others and instrument/apparatus) have both their limitations [78]. The best methods of assessment are multimodal and multimethod. However, such an analysis is not always possible in a clinical setting. Once again, we need to be cautious when interpreting the results. 

In conclusion, evidence supporting the psychometric properties of questionnaires which are currently available to measure fear of pain in patients with musculoskeletal pain is still incomplete. Future research on the validity of fear of pain measures seems warranted. In order to facilitate the clinical application of these instruments, it is recommended that the focus of research be on an agreement of the conceptual and operational definitions of the various constructs. One way of starting that process is to combine the more established psychometric procedures with a qualitative approach, in order to be able to incorporate the patient's perspective. 

## Figures and Tables

**Table 1:** A simplified summary of the criteria for assessing the levels of reliability, validity, and responsiveness adjusted after Wind et al. [21].

Reliability	Ways to measure	Criteria	Level
Stability over time (i) test-retest	Pearson product moment correlation coefficient	Low^a^	
	Spearman correlation coefficient	Low^a^	
	Percentage of agreement %	High	% > 0.90 and the raters can choose from more than two score levels
		Moderate	% > 0.90 and the raters can choose from between two score levels
		Low	The raters can choose only between two score levels
	Kappa value	High	*κ* > 0.60
		Moderate	0.41 < *κ* < 0.60
		Low	*κ* < 0.60
	Intraclass correlation coefficient (ICC)	High	ICC > 0.90
		Moderate	ICC 0.75–0.90
		Low	ICC < 0.75

Internal consistency	Cronbach's alpha	High	*α* > 0.80
		Moderate	0.71 < *α* < 0.80
		Low	*α* > 0.70
	Intraclass correlation coefficient (ICC)	High	ICC > 0.90
		Moderate	ICC 0.75–0.90
		Low	ICC < 0.75

VALIDITY Content/Face		High	The test measures what it is intended to measure and all relevant components are included
		Moderate	The test measures what it is intended to measure but not all relevant components are included
		Low	The test does not measure what it is intended to measure

Criterion-related (i) concurrent (ii) predictive		High	Substantial similarity between the test and the criterion measure (with documented high reliability and validity)
			(PA ≥ 90%; *κ* > 0.60; *r* ≥ 0.75)
		Moderate	Some similarity between the test and the criterion measure
			(PA ≥ 70%; *κ* ≥ 0.40; *r* ≥ 0.50)
		Low	Little or no similarity between the test and the criterion measure (PA < 70%; *κ* < 0.40; *r* < 0.50)

Construct (i) convergent (ii) divergent		High	Good ability to differentiate between groups or interventions, or good convergence/divergence between similar tests (*r* > 0.95^b^)
		Moderate	Moderate ability to differentiate between groups or interventions, or moderate convergence/divergence between similar tests (0.85 < *r* ≤ 0.95^b^)
		Low	Poor ability to differentiate between groups or interventions, or weak convergence/divergence between similar tests (*r* ≤ 0.85^b^)

Responsiveness	Effect size	High	*r * ^ c^, SMR^d^≥ 0.80
		Moderate	0.40 < *r * ^c^, SMR^d^< 0.80
		Low	*r * ^ c^, SMR^d^< 0.40
	Area under ROC-curve (AUC)	High	AUC > 0.75
		Moderate	0.5 ≤ AUC ≤ 0.75
		Low	AUC < 0.5

^a^According to Grotle et al. [27] ^b^The correlations are between two random variables, therefore the criteria are higher compared to those chosen by Wind et al. The original limit of *r* = 0.50 gives an explained variation of less than 13%; *r* = 0.85 implies that 53% of the variation of the variable at one time point is explained by knowing the other time point, and *r* = 0.95 implies an explained variation of 69%. Formula for calculation; Var⁡(*Y* | *x*) = (1 − *ρ* ^2^)*σ*_*Y*_^2^, where both *X* and *Y* are random variables see also p. 484 in [28]. ^c^Effect size according to Wind et al. [21] Correlation (pearson) between test results preoperative and postoperative, and also pretreatment and posttreatment. ^d^Cohen's effect size. Criteria limits defined according to Grotle et al. [27].

**Table 2:** A brief summary of the assessment of the psychometric properties of all selected questionnaires presented in alphabetical order.

Instrument	Reliability		Validity			Responsiveness
	Internal consistency	Reproducibility	Face content	Construct (i) convergent (ii) divergent	Criterion related (i) concurrent (ii) predictive	(i) Effect size (ii) Area under the curve (AUC)

Fear-Avoidance Beliefs Questionnaire (FABQ)						

*Chinese* Lee et al. [29, 30]	FABQ = high	FABQ = moderate	Content: Unevaluated methods^a^	Construct: Unevaluated methods^a^	Concurrent FABQ_total _= low-moderate FABQ_pa_, FABQ_work_, FABQ_prognosis work _= low	Effect size: FABQ_work_ = low FABQ_pa_ = low

*Dutch* Crombez et al. [31]; Reneman et al. [32]	FABQ_work_ = high FABQ_pa _= low				Concurrent FABQ_work_, FABQ_pa_ = low-moderate Predictive: Unevaluated methods^a^	

*English* Waddell et al. [33]; McCracken et al. [34]	FABQ_work_ = high FABQ_pa _= moderate	FABQ = high FABQ_work_, FABQ_pa _= low		Construct: Unevaluated methods^a^	Concurrent FABQ_work_, FABQ_pa_, FABQ_total_ = low moderate Predictive: Unevaluated methods^a^	

*French* Chaory et al. [35]		FABQ_work_ = moderate FABQ_pa_ = low		Construct: Unevaluated methods divergent FABQ_work_, FABQ_pa_ = low		Effect size: FABQ_work_ = low FABQ_work_ = low

*German* Pfingsten et al. [36]; Staerkle et al. [37, 38],	FABQ_work _= high FABQ_pa _= high (after FA) FABQ_total_; FABQ_work1_; FABQ_work2_ = high FABQ_pa_ = low	FABQ_work total_ = low FABQ1, FABQ_work2_ = low FABQ_pa_ = low FABQ_work_ = high FABP_pa_ = moderate		Construct: Unevaluated methods^a^	Concurrent FABQ_work_ (base); FABQ_pa _(base) = low-moderate FABQ_work_ (followup) = low FABQ_pa_ (followup) = low Predictive: Unevaluated methods^a^	Effect size: FABQ_work _= low FABQ_pa _= moderate

*Greece* Georgoudis et al. [39]	FABQ_pa_ = moderate FABQ_work1_, FABQ_work2 _= high	FABQ_pa_ = moderate FABQ_work1_, FABQ_work2_ = high		Convergent: FABQ_work_, FABQ_pa_ = low Divergent: FABQ_work1_, FABQ_pa_ = low		

*Hebrews* Jacob et al. [40]	FABQ_work_ = high FABQ_pa_ = low	FABQ_work_, FABQ_p_; FABQ_work_ = moderate FABQ_pa_ = high				

*Norwegian* Grotle et al. [27]	FABQ_work_ = high FABQ_pa_ = moderate	FABQ_work_ = moderate FABQ_pa_ = low		Construct validity: FABQ_work chronic_; FABQ_pa chronic_ = low Unevaluated methods^a^		Effect size: FABQ_work_ = moderate FABQ_pa_ = low

*Spanish* Kovacs et al. [41]	FABQ = high	FABQ = high		FABQ_total_, FABQ_work,_ FABQ_pa_ = low		Floor ceiling (i) Unevaluated methods^a^

Fear Avoidance of Pain Questionnaire (FAPQ)						

*English* Crowley and Kendall [42]	FAPQ = High	FAPQ = Low			Concurrent FAPQ = low moderate	Sensitivity and specificity (i) Unevaluated methods^a^

Fear of Pain Questionnaire (FPQ)						

*English* Hursey and Jacks [43]; McCracken et al. [34]; McNeil and Rainwater [44, 45]	Total scale = moderate-high Minor = moderate-high Severe = high Medical = low-high				Concurrent: FPQ_total_ = low Unevaluated methods^a^	

Pain Anxiety Symptoms Scale (PASS)						

*Dutch *Crombez et al. [30]	PASS = high			Convergent PASS = Low-moderate		

*Combined Dutch and USA* study Roelofs et al. [46] Dutch/The Netherlands English/USA Original PASS + PASS-20	PASS_total_, PASS_cognitive anxiety_, PASS_fear of pain_, PASS_phys.s of anxiety_, PASS-20 = high PASS_escape/avoidance_ = moderate PASS_escape/avoidance_, PASS_physiological symptoms of anxiety_ = moderate			Convergent PASS_total_, PASS_cognitive anxiety_ PASS_fear of pain_, PASS_escape/avoidance_, PASS_physiological symptoms of anxiety_: PASS-20_cognitive anxiety_, PASS-20_fear of pain_, PASS-20_escape/avoidance_, = low		

*English 53 items* McCracken et al. [18, 34]; Burns et al. [47]; Strahl et al. [48]	PASS_total_, PASS_somatic,_ PASS_cognitive_; PASS_fear_, PASS_escape/avoidance _= high				Concurrent PASS_total_, PASS_somatic_, PASS_fear_, and PASS_cognitive_ = low-moderate PASS_escape/avoidance_ = low	

*English or French 20 items *Coons et al. [49]	PASS20 = high PASS20_escape/avoidance_ = low PASS20_physiological symptoms of anxiety_ = moderate PASS20_cognitive anxiety_, PASS20_fear of pain_ = high		PASS20_fear_ = low PASS20_escape_, PASS20_physiological_, PASS20_cognitive_, and = low-moderate			Effect size PASS20; PASS20_fear_; PASS20_escape_, PASS20_physiological_, PASS20_cognitive_ = moderate

*English* Larsen et al. [28]			PASS_catastrophic thoughts_, PASS_physiological anxiety_, PASS_escape/avoidance_, PASS_cognitive interference_, and PASS_coping strategies _= low Unevaluated methods^a^			

Tampa Scale for Kinesiophobia (TSK)						

*Dutch 12 items* Vlaeyen et al. [50]	TSK_17_ = moderate (17 items) TSK_13_ = low moderate (13 items) Item-total: Unevaluated methods^a^			Construct: Unevaluated methods^a^	Concurrent: TSK_total_, TSK_fear_ = low moderate TSK_harm_, TSK_Avoidance of activity_ = low	

*Dutch 13 items* Goubert et al. [51]	TSK_total_ CLBP = moderate; FMS = high TSK_activity avoidance _CLBP; FMS = moderate. TSK_pathologic somatic focus _CLBP; FMS = low			Construct: Unevaluated methods^a^		

*Dutch 11 items *Roelofs et al. [52]	UED TSK_11_ = moderate Chronic pain TSK_11_ = moderate			Construct: Unevaluated methods^a^	Concurrent TSK_SF_ = low TSK_AA_ = low	

*TSK English* 4 items Burwinkle et al. [53]	TSK_4_ = moderate			Construct: Unevaluated methods^a^		

*English* 11 items Woby et al. [54]	TSK_total_, TSK_11 _= moderate Item-total Unevaluated methods^a^	TSK = moderate	TSK_total_:, TSK_11_: = low			Effect size: TSK_total_, TSK_11_ = low AUC TSK_total_, TSK_11_ = moderate Sensitivity and specificity-Unevaluated methods^a^

*English 13 items* Clark et al. [55]; French et al. [56]	TSK_total _= high TSK_taa_ = moderate TSK_tsf _= low Item-total Unevaluated methods^a^			Construct: Unevaluated methods^a^	Concurrent low	

*Norwegian* Damsgård et al. [57]		Unevaluated methods^a^		Construct: Unevaluated methods^a^		

*Swedish 17 items* Lundberg et al. [7]; Bunketorp et al. [58]	TSK = high Item-total Unevaluated methods^a^	TSK = low-high Unevaluated methods^a^	Face and content: Unevaluated methods^a^	Construct: Unevaluated methods^a^		

Abbreviations in reliability: *α*: Cronbach's alpha, ICC: Intraclass Correlation Coefficient, r_p_: Pearson's product moment correlation coefficient, RC, RP; RV.

Abbreviations in validity: CFA: Confirmatory Factor Analysis, EFA: Exploratory Factor Analysis.

Abbreviations in responsiveness: AUC: Area Under Curve, SRM: Standardised Response Mean.

^a^Additional methods not evaluated as they are not included in the table of modified Wind criteria.

**Table 3:** Overview of psychometric properties of all selected questionnaires.

Instrument	Study population	Reliability	Validity	Responsiveness
Presented in accordance with language version, in alphabetical order	(a) sample size (b) gender (% male) (c) mean age (years) (d) pain duration (months)	(a) internal consistency (b) reproducibility (c) other	(a) content (b) construct (1) convergent (2) divergent (c) criterion related (1) concurrent (2) predictive (d) other	(a) Effect size (b) Area under ROC- curve (AUC) (c) other

Fear-Avoidance Beliefs Questionnaire (FABQ)				

*Chinese* Lee et al. [29, 30]	(a) 476 NP (b) 40% (c) 42.5 (d) no info	(a)***α*** FABQ = 0.90 (b) **ICC** FABQ = 0.81 (c) **Item-total** FABQ = 0.31–0.68	(a) **Content:** Translation and back translation + Expert group (b) **Construct:** PCA: 3 factors (61.6%) (c) **Criterion related:** Concurrent **r**_****s****_ FABQ_total_ (NRS) entry = 0.34***; discharge = 0.33***; (NPQ) entry = 0.56***; discharge = 0.53***; (SF36 physical) entry = −0.45***; discharge = −0.64***; (SF36 mental) entry = −0.36***; discharge = −0.43*** FABQ_pa_ *pain related:* pain rating = −0.32**; Northwick Park Neck Pain Quest. = −0.39** *physical:* active range of motion = −0.13**; age = −0.12**; strength index = −0.15**; SF36 physical = −0.47** *emotional:* SF36: mental = −0.12** FABQ_work _ *pain related: *pain rating = −0.44**; Northwick Park Neck Pain Quest. = −0.47** *physical:* age = −0.07**; active range of motion = −0.19**; SF36: physical = −0.40**; strength index = −0.17** *psychosocial:* SF36: mental = −0.36** FABQ_prognosis work_ *pain related:* Northwick Park Neck Pain Quest. = −0.422**; pain rating = −0.35** *physical:* age = −0.12**; active range of motion = −0.20**; strength index = −0.17** *psychosocial:* SF-36: mental = −0.29**; SF-36: physical = −0.49**	(a) SRM FABQ_work_ = 0.38 FABQ_pa_ = 0.32 (b) —

*Dutch* Crombez et al. [31]; Reneman et al. [32]	(a) 35 CP + 38 CLBP; 58 CLBP; (b) 31% + 34%; 67% (c) 36.1+ 40.8; 35.6 and 40.4 (d) 80.4+76; no info	(a)***α*** FABQ_work_ = 0.84 FABQ_pa_ = 0.57 (b) —	(a) — (b) — (c) **Criterion related: Concurrent** FABQ_work _(r_p_) *pain related:* baseline pain = 0.56***; disability (RDQ) = 0.63***; experienced pain increase = 0.42** *physical:* FCE-dynamic forward bend = 0.30*; FCE-lifting performance (m_en_) = −0.37* peak torque = 0.45** *psychosocial:* kinesiophobia (TSK) = 0.56*** + 0.53**; negative affect (NEM) = 0.35* + 0.38** FABQ_pa_ *pain related:* base-line pain = 0.31*; disability (RDQ) = 0.51*** *physical:* FCE-static forward bend = 0.30*; peak torque = −0.45** *psychosocial:* kinesiophobia (TSK) = 0.57***, 0.76***; 0.392; negative affect (NEM) = 0.42** ** Regression** Dependent: Disability (RDQ) *β* FABQ_work_ = 0.57**; *β* FABQ_pa_ = 0.40* (d) —	(a) — (b) —

*English* Waddell et al. [33]; McCracken et al. [34]	(a) 184 LBP; 45 CP (b) 55%; 47% (c) 39.7; 46.3 (d) 13.7; 27	(a)***α*** FABQ_work _= 0.88 FABQ_pa _= 0.77 (b) **r**_****p****_ FABQ_work_ = 0.95 FABQ_pa _= 0.88 (c) **Other: kappa** FABQ = 0.74***	(a) — (b) **Construct** PCA: *2 factors:* FABQ_work beliefs (work)_ and FABQ_activity beliefs (pa)_ (c) **Criterion related: Concurrent** R_p _FABQ_work _ avoidance (PBC) = 0.35**; depressive symptoms (ZDI) = 0.41***; disability (RDQ) = 0.55***; help seeking (PBC) = 0.43**,(PDI) = 0.48** pain severity (VAS) = 0.23**; psych.distress (MSPQ) = 0.36***; work loss_present_ = 0.39***; work loss_past year_ = 0.55*** FABQ_pa_ avoidance (PBC) = 0.46**; depressive symptoms (ZDI) = 0.36***; disability (RDI) = 0.51***; (PDI) = 0.37*; work loss^past year^ = 0.23** FABQ_total_ avoidance (PBC) = 0.46**; disability (PDI) = 0.52***; help seeking (PBC) = 0.46** **Regression** (1) Dependent: disability (RDQ); (PDI) ß FABQ_work_ = 0.32***; 0.26**; ß FABQ_pa_ = 0.27*** (2) Dependent: work loss_past year_ ß FABQ_work_ = 0.48*** (3) Dependent: help seeking ß FABQ_work_ = 0.43* (d) —	(a) — (b) —

*French* Chaory et al. [35] (3 studies in 1 article)	(a) 31 CLBP; 147 CLBP; 70 CLBP (b) no info; no info; no info (c) 46; 45.3; 42.5 (d) 42.5; 113; 45.5	(a) — (b) **ICC** FABQ_work_ = 0.88 FABQ_pa_ = 0.72	(a) — (b) **Construct: Factor Analysis (FA):** 4 factors; fear of professional activities, physical activity, beliefs about return to work, beliefs about the responsibility of work with chronic symptoms *2 factors*; beliefs about professional activities, beliefs about physical activity **Construct: divergent** FABQ_work_: depression (HADS) = 0.29; disability (Quebec score) = 0.36 FABQ_pa_: handicap (VAS) = 0.36; 0.34	(a) FABQ_work_ = 0.30 FABQ_work_ = 0.31 (b) —

*German* Pfingsten et al., [36]; Staerkle et al., [37, 38]	(a) 302 CLBP; 388 LBP (b) 52%; 44% (c) 44.6; 54.2 (d) 71.4; no info	(a)***α*** FABQ_work _= 0.89 FABQ_pa _= 0.94; 0.91 ***α* (after FA)** FABQ_total_ = 0.91; FABQ_work1_ = 0.89; FABQ_work2_ = 0.94 FABQ_pa_ = 0.69 (b) r_p_ FABQ_work_ = 0.89 FABQ_work1 _= 0.83; FABQ_work2_ = 0.89; FABQ_pa_ = 0.90; 0.84 (c) Weighted Kappa = 0.76** **ICC** FABQwork = 0.91 FABPpa = 0.83	(a) — (b) **Construct: Factor Analysis** *3 factors*: work as a cause (43.4%); prognosis work (11.8%) physical activity (8.9%) *2 factors* (exclusion item 1, 8, 13, 14): fear-avoidance beliefs relation work-LBP; fear-avoidance beliefs relation physical activity-LBP (c) **Criterion related: Concurrent (r_**p**_)** FABQ_work_(baseline) *pain related:* disability (RDQ) = 0.57***; pain severity (VAS) = 0.47*** *physical:* work absence last month = 0.47*** *psychosocial:* depressive symptoms (ZDI) = 0.42***; psychol. distress (MSPQ) = 0.36*** FABQ_pa_ (baseline) *pain related:* disability (RDQ) = 0.56***; pain severity (VAS) = 0.48*** *physical:* work absence last month = 0.42*** *psychosocial:* depressive symptoms (ZDI) = 0.37***; psychological distress (MSPQ) = 0.31^∗∗∗2^ FABQ_work_(followup) *Physical:* disability (RDQ) = 0.47***; work absence last month = 0.44*** *Psychosocial:* depressive symptoms (ZDI) = 0.34***; pain severity (VAS) = 0.37*** psychological distress (MSPQ) = 0.29*** FABQ_pa_ (followup) *pain related:* disability (RDQ) = 0.39***; pain severity (VAS) = 0.29*** *physical:* work absence last month = 0.29*** *psychosocial:* depressive symptoms (ZDI) = 0.25***; psychological distress (MSPQ) = 0.24*** **Regression** (1) Dependent: disability (FFbH-R) *β* FABQ_work_ = 0.35** *β* FABQ_pa_ = 0.22* (2) Dependent: work loss *β* FABQ_work_ = 0.65*** *β* FABQ_pa_ = 0.12* (d) —	(a) Effect size FABQ_work _= 0.33 FABQ_pa _= 0.41 (b) —

*Greece* Georgoudis et al. [39]	(a) 70 CLBP (b) 17% (c) 42.2 (d) no info	(a) *α* FABQ_pa_ = 0.72 FABQ_work1_ = 0.90 FABQ_work2_ = 0.86 (b) ICC FABQ_work1_ = 0.93 FABQ_work2_ = 0.94 FABQ_pa_ = 0.85	(a) — (b) **Construct: Convergent** FABQ_work_ = TSK = 0.39***; FABQ_pa_ = TSK = 0.55*** **Divergent** FABQ_work1_: HADS = 0.26*; HADS_depression_ = 0.35*; HADS_anxiety_ = 0.22*; Pain intensity (VAS) = 0.56 FABQ_pa_ = HADS_depression_ = 0.29*; Pain-intensity (VAS) = 0.49	(a) — (b) —

*Hebrews* Jacob et al. [40]	(a) 151 LBP (b) 44% (c) 44.3 (d) >1	(a) ***α*** FABQ_work_ = 0.89 FABQ_pa_ = 0.70 (b) **ICC** FABQ_work_ = 0.90 FABQ_pa_ = 0.77 **Kappa** FABQ_work_ = 0.53 FABQ_pa_ = 0.64	(a) — (b) — (c) —	(a) — (b) —

*Norwegian* Grotle et al. [27]	(a) (123 acute LBP)+ 50 CLBP (b) 45% + 38% (c) 37.8+ 40.4 (d) 0.3 + 18.7	(a) ***α***(n = 28) FABQ_work_ = 0.90 FABQ_pa_ = 0.79 (b) **ICC** FABQ_work_ = 0.82 FABQ_pa_ = 0.66	(a) **Content:** Translation-back translation + Pilot study (b**) Construct: Confirmatory Factor Analysis (CFA):** *2 factors * (item 8 excluded); FABQ_work _(60%), FABQ_pa _(54%) FABQ_work chronic_ *pain related:* disability (ODI) = 0.34*; disability days = 0.52** *physical:* age = −0.36*; smoking = 0.52**; straight leg raising (SLR) = −0.31* *psychosocial:* (HSCL25) = 0.43**; (MSPQ) = 0.59**; nonorganic signs = 0.32* FABQ_pa chronicp_ *pain related:* disability (ODI) = 0.39**; disability days = 0.33* * physical:* smoking = 0.45**; straight leg raising = −0.36*; walking test = 0.31* *psychosocial:* (MSPQ) = 0.32*; (HSCL25) = 0.36* (c) —	(a) SRM (Cohen's effect size) FABQ_work_ = 0.56 FABQ_pa_ = 0.32 (b) —

*Spanish* Kovacs et al. [41]	(a) 209 LBP (b) 42% (c) 45.7 (d) 65	(a) ***α*** FABQ = 0.94 (b) **ICC** FABQ = 0.97 (c) **Weighted kappa** FABQ = 0.74	(a) — (b) (c) **Criterion related: Concurrent** FABQ_work_ Disability (RM) = 0.467***; low back pain (VAS) = 0.381***; quality of life (SF-36, PCS) = −0.386***; quality of life (SF-36, MCS) = −0.320**; referred pain (VAS) = 0.323*** FABQ_pa _ disability (RM) = 0.412***; low back pain (VAS) = 0.324***; quality of life (SF-36, PCS) = −0-304***; quality of life (SF-36, MCS) = −0.201**; referred pain (VAS) = 0.251*** FABQ_total_ Disability (RM) = 0.522***; low back pain (VAS) = 0.398***; quality of life (SF-36, PCS) = −0432***; quality of life (SF-36, MCS) = −0.361*; referred pain (VAS) = 0.320***	(a) — (b) — (c) Floor and ceiling effects FABQ_work_ Floor effect 2.9% Ceiling effect 8.6% FABQ_pa_ Floor effect 1.4% Ceiling effect 23.9% FABQ_total_ Floor effect 1.0% Ceiling effect 3.3%

Fear Avoidance of Pain Scale (FAPS)				

*English* Crowley and Kendall [42]	(a) 21 + 63 chronic musculoskeletal pain (b) 33% (c) 35.7 (d) 48.8	(a)***α*** 0.92 (b) **r_**p**_** 0.84** (*N* = 21)	(a) — (b) — (c) **Criterion related: Concurrent** depression (Zung) = 0.48** −0.54**; disability (FLP) = 0.42** −0.69**; impact of pain (MPI) = −0.04* −0.73**; pain (VAS) = 0.37** −0.48**; pain-related thoughts (PRSS-CAT) = 0.51** −0.63**	(a) — (b) — (c) Sensitivity to treatment Change ANOVA Pretreatment versus post-treatment 14.85* Pre-treatment versus followup 22.4*

Fear of Pain Questionnaire (FPQ)				

*English* Hursey and Jacks [43]; McCracken et al. [34]; McNeil and Rainwater [44], [45]	(a) 45 headache and 58 controls,45 CP; 40 CP and 40 healthy controls +275 students (b) 12%; 47%; 32.5% (c) 462; 474 (d) 272; no info	(a) ***α*** Headache: FPQ_Total scale_ = 0.92 FPQ_Minor_ = 0.86 FPQ_Severe_, FPQ_Medical_ = 0.87 Controls: FPQ_Total scale_ = 0.80 FPQ_Minor_ = 0.71 FPQ_Severe_ = 0.86 PQ_Medical_ = 0.64 (b) —	(a) — (b) — (c) **Criterion related: Concurrent** FPQ_total_ disability (PDI) = 0.15; somatic = 0.38*** anger = 0.13; anxiety = 0.0.37***; avoidance (PBC) = 0.25; complaints (PBC) = 0.22; depression = 0.46***; help seeking (PBC) = 0.11; pain (MPQ) = 0.03 FPQ_Severe_ Complaints (PBC) = 0.32** (d) *Intercorrelations subscales* r_*α*_ Minor-medical = 0.30***; Severe-medical = 0.65*** *Group effect for: * Severe pain < 0.024; Gender effect: Subscales and total scale *P* < 0.01, Ethnical effect in control group, Medical pain *P* < 0.05	(a) — (b) —

Pain Anxiety Symptoms Scale ( PASS)				

*Dutch* Crombez et al. [30]	(a) 31 CLBP (b) 48% (c) 41.61 (d) 121	(a) ***α*** PASS = 0.91 (b) —	(a) — (b) — (c) **Criterion related: Concurrent validity** catastrophizing (PCS) = 0.61***; kinesiophobia (TSK) = 0.34*; negative affect (NEM) = 0.63***; weight lifting (s) = −0.33*	(a) — (b) —

*Combined Dutch and USA study* Roelofs et al. [46] Dutch/The Netherlands English/USA Original PASS + PASS-20	(a) 398 FMS (Dutch)+ 2. 228 CLBP (Dutch) + 3. 284 pain (USA) (b) 6% + 39% + 35% (c) 47.6 + 50.0 + 46.6 (d) 159 + 2. 74 + 63	(a) ***α*** PASS_total_ = 0.94 PASS_cognitive anxiety_ = 0.85 PASS_fear of pain_ = 0.86 PASS_escape/avoidance_ = 0.75 PASS_phys.s of anxiety_ = 0.86 PASS-20 = 0.91 PASS_cognitive anxiety_ = 0.84 PASS_fear of pain_ = 0.84 PASS_escape/avoidance_ = 0.73 PASS_physiological symptoms of anxiety_ = 0.74 (b) —	(a) — (b) **Construct validity: Convergent validity** PASS_total_: catastrophizing (PCS) = 0.76; fear (TSK) = 0.55, PVAQ = 0.59 PASS_cognitive anxiety_: catastrophizing = 0.69; fear (TSK) = 0.45; PVAQ = 0.52 PASS_fear of pain_: catastrophizing = 0.73; fear (TSK) = 0.54; PVAQ_n_ = 0.51 PASS_escape/avoidance_: catastrophizing = 0.58; fear (TSK) = 0.49; PVAQ = 0.55 PASS_physiological symptoms of anxiety_: catastrophizing (PCS) = 0.76; fear (TSK) = 0.41; PVAQ = 0.42 PASS-20_cognitive anxiety_: catastrophizing (PCS) = 0.70; fear (TSK) = 0.44; PVAQ = 0.54 PASS-20_fear of pain_: catastrophizing (PCS) = 0.74, fear (TSK) = 0.53; PVAQ = 0.53 PASS-20_escape/avoidance_; catastrophizing (PCS) = 0.54; fear (TSK) = 0.47; PVAQ = 0.50 PASS_physiological symptoms of anxiety_: catastrophizing (PCS) = 0.56; fear (TSK) = 0.37; PVAQ = 0.40 (c) —	(a) — (b) —

*English 53 items* McCracken et al., [18]; [34]; Burns et al., [47]; Strahl et al. [48]	(a) 104 CP; 45 CP; 98 CP; 154 RA (b) 46%; 47%; 100%; 14% (c) 45; 46.3; 38.9; 54 (d) 63.; 27; 9.5; 178	(a) ***α*** PASS_total_ = 0.941 PASS_somatic_ = 0.891 PASS_cognitive_ = 0.871 PASS_fear_ = 0.851 PASS_escape/avoidance _= 0.811 (b) —	(a) — (b) — (c) **Criterion related: Concurrent validity** **r_**p**_** PASS_total_ *pain related:* disability (PDI) = 0.45***; 0.61***; pain interference (IS) = 0.22**subjective pain (McGill)_sensory _= 0.31***; (McGill)_affective _= 0.44***; (MPI)_pain severity _= 0.32***; pain experience (MPQ)_pain_ = 0.56***; pain severity (PSS) = 0.31***; subjective pain (MPI)_interference_ = 0.39*** *physical:* carrying capacity (CCE) = −0.30**; general activity (GAS) = −0.23**; lifting capacity (PILE) = −0.30**; tranquilizer = 0.29** *psychosocial:* anxiety (STAI)_trait_ = 0.60***; 0.54***; coping (CSQ)_catstrophising _= 0.73***; depression (BDI) = 0.57***; 0.52***; pain behaviour (PBC)_avoidance e_ = 0.45**; pain behaviour (PBC)_helpseeking_ = 0.32*; symptoms of anxiety (CSAQ)_cognitive _= 0.54***; symptoms of anxiety (CSAQ)_somatic _= 0.61*** PASS_somatic_ *pain related:* disability (PDI) = 0.39***; 0.51***; pain experience (MPQ)_pain _= 0.63***subjective pain (McGill)_sensory _= 0.45***; (McGill)_affective _= 0.51***; (MPI)_pain severity _= 0.35***; (MPI)_interference _= 0.28** *physical:* tranquilizer = 0.25** *psychosocial:* anxiety (STAI)_trait_ = 0.52***; coping (CSQ)_catstrophising_ = 0.67***; depression (BDI) = 0.51***; symptoms of anxiety (CSAQ)_cognitive _= 0.49***; (CSAQ)_somatic _= 0.74***	(a) — (b) —
			PASS_cognitive_ *pain related:* disability (PDI) = 0.39***; 0.51***; pain experience (MPQ)_pain _= 0.43**; subjective pain (McGill)_affective _= 0.33***; (McGill)_sensory _= 0.26**; subjective pain (MPI)_interference _= 0.25**; (MPI)_pain severity _= 0.35*** *Physical:* tranquilizer = 0.27** *Psychosocial:* anxiety (STAI)_trait_ = 0.67***; coping (CSQ)_catstrophising_ = 0.67***; depression (BDI) = 0.67***; pain behaviour (PBC)_avoidance_ = 0.33*; symptoms of anxiety(CSAQ)_cognitive _= 0.61***; (CSAQ)_somatic _= 0.55*** PASS_fear_ *pain related:* disability (PDI) = 0.30***; 0.38**; pain experience (MPQ)_pain_ = 0.44**; subjective pain (McGill)_affective _= 0.36***; (MPI)_interference _= 0.31***; (MPI)_pain severity _= 0.28** *psychosocial:* anxiety (STAI)_trait_ = 0.53***; depression (BDI) = 0.50^∗∗∗1^; coping (CSQ)_catstrophising_ = 0.66***; pain behaviour (PBC)_avoidance_ = 0.39**; (PBC)_complaints_ = 0.39**; symptoms of anxiety (CSAQ)_cognitive _= 0.53***; (CSAQ)_somatic _= 0.56*** PASS_escape/avoidance_ *pain related:* disability (PDI) = 0.30***; subjective pain (McGill)_affective _= 0.31***; (MPI)_interference _= 0.36*** *Psychosocial:* anxiety (STAI)_trait_ = 0.29**; coping (CSQ)_catstrophising_ = 0.42***; depression (BDI) = 0.30*** (d) **Prediction: Regression** (1) Dependent: Interference1: *β* PASS_total _(+STAI-T) = 0.28*; 0.087*; (+MPQ-sensory) = 0.39***; (+Pain severity) = 0.28**; (+PSS) = 0.254***; (+BDI) = 0.254*** (2) Dependent: Disability: *β* PASS_total _(+BDI) = 0.32*; (+STAI-T) = 0.38**; (+MPQ-sensory) = 0.44***; (+Pain severity) = 0.32*** (3) Dependent: General activity: *β* PASS (+STAI) = 0.087*; (+BDI) = 0.057*; (+PSS) = 0.054* (4) Dependent: Pain severity (MPQ); *β* (PASS_physiological_) = 0.63*** (5) Dependent: Disability (PDI); *β* PASS_escape/avoidance)_ = 0.44** (6) Dependent: Avoidance (PBC); *β* PASS_escape/avoidance_ = 0.54*** (7) Dependent: Complaints (PBC); *β* PASS_fear_ = 0.39** (8) Dependent: Help seeking (PBC); *β* PASS_escape/avoidance_ = 0.30	

*English or French 20 items* Coons et al. [49]	(a) 201 current pain (b) 45% (c) 41.5 (d) 23	(a) ***α*** PASS = 0.83 PASS_fear of pain_ = 0.83 PASS_escape/avoidance_ = 0.70 PASS_ physiological symptoms of anxiety_ = 0.76 PASS_cognitive anxiety_ = 0.85 (b) —	(a) — (b) Construct validity: Effect size PASS20_fear_ *pain related:* disability (MPI) = 0.32**; pain severity (MPI) = 0.28** *physical:* physiological (ASI) = 0.35** *psychosocial:* cognitive (ASI) = 0.37**; public (ASI) = 0.31** Affect (AIMS2) = 0.43*; pain sx (AIMS2) = 0.22*; Social (AIMS2) = 0.33*; Symptoms (ASE) = −0.38*; pain (ASE) = −0.45*; negative affect (MPI) = 0.44**; perceived control (MPI) = −0.34**; social support (MPI) = 0.18**; Passive coping = 0.73*; Active coping = −0.44* PASS20_escape_ *pain related * disability (MPI) = 0.36**; pain severity (MPI) = 0.16*; pain (ASE) = −0.30*; symptoms (ASE) = −0.29* *physical * physiological (ASI) = 0.23** *psychosocial * Affect (AIMS2) = 0.26*; Role (AIMS2) = 0.20*; Social (AIMS2) = 0.24*; cognitive (ASI) = 0.28**; public (ASI) = 0.26**; negative affect (MPI) = 0.22**; social support (MPI) = 0.19**; Active coping = −0.39*; Passive coping = 0.67* PASS20_physiological_ *pain related * disability (MPI) = 0.27**; pain (ASE) = −0.35*; pain severity (MPI) = 0.15*; perceived control (MPI) = −0.23**; symptoms (ASE) = −0.29* *physical * physiological (ASI) = 0.33** *psychosocial * affect (AIMS2) = 0.51*; pain sx (AIMS2) = 0.25*; role (AIMS2) = 0.29*; social (AIMS2) = 0.31*; cognitive (ASI) = 0.34**; public (ASI) = 0.26**; Active coping = −0.28*; Passive coping = 0.61*; negative affect (MPI) = 0.44** PASS20_cognitive_ *pain related * Disability (MPI) = 0.25**; Pain (ASE) = −0.42*; pain severity (MPI) = 0.20** Symptoms (ASE) = −0.32* *Physical * Physiological (ASI) = 0.32** *psychosocial * affect (AIMS2) = −0.42*; cognitive (ASI) = 0.31**; public (ASI) = 0.25**; negative affect (MPI) = 0.39**; perceived control (MPI) = −0.30**; Active coping = −0.33*Passive coping = 0.69*; social (AIMS2) = 0.30* **Regression** (1) Dependent: Affect; PASS and STAI = 0.332*** (2) Dependent: Role function; PASS and STAI = 0.315** (d) —	(a) **Effect size** r_p_ 0.68*** (b) —

*English* Larsen et al. [28]	(a) 259 CP (b) 58% (c) 38.2 (d) 21	(a) — (b) —	(a) — (b) **Construct validity: EFA (PCA)** *5-factor solution* (44%): catastrophic thought; physiological anxiety; escape/avoidance; cognitive interference; coping strategies **r_**p**_** PASS_catastrophic thoughts_ *pain related:* pain (MPQ)_affective scale_ = 0.23** *psychosocial:* anxiety (STAI) = 0.49**; depression (BDI) = 0.40** PASS_physiological anxiety _ *pain related:* pain (MPQ)_present pain index_ = 0.29** *physical:* analgesic use = −0.18* *psychosocial:* anxiety (STAI) = 0.35**; depression (BDI) = 0.35**; pain (MPQ)_affective scale_ = 0.33**; (MPQ)_pain intensity_ = 0.30**; (MPQ)_sensory scale_ = 0.36**; pain-related change in lifestyle = 0.17*; pain-related distress = 0.22** PASS_escape/avoidance_ *pain related:* Pain (MPQ)_present pain index_ = 0.25**; (MPQ)_pain intensity_ = 0.24** *physical:* analgesic use = −0.25** *psychosocial:* anxiety (STAI) = 0.30**; depression (BDI) = 0.19**; pain-related change in lifestyle = 0.38**; pain-related distress = 0.25** PASS_cognitive interference_ *pain related:* pain (MPQ)_present pain index_ = 0.35** *physical: *analgesic use = −0.33** *psychosocial:* anxiety (STAI) = 0.50**; depression (BDI) = 0.38**; pain-related change in lifestyle = 0.25**; pain-related distress = 0.21**; pain (MPQ)_affective scale_ = 0.17**(MPQ)_pain intensity_ = 0.22** PASS_coping strategies_ * physical:* analgesic use = −0.27** *psychosocial:* anxiety (STAI) = 0.33**; depression (BDI) = 0.20**	(a) — (b) —

Tampa Scale for Kinesiophobia (TSK)				

*Dutch 12 items* Vlaeyen et al. [50]	(a) 129 + 33 CLBP (b) 39% + 48% (c) 40.1 + 37.4 (d) 119 + 91	(a) ***α*** TSK_17_ = 0.77 (17 items) TSK_13_ = 0.53–0.73 (13 items) (b) — (c) Item total <.40	(a) — (b) **Construct validity: PCA:** *4 factors* (36.2%): harm, fear of (re)injury, importance of exercise, avoidance of activity (c) **Criterion-related validity:** Concurrent validity: TSK_total_: catastrophising (PCS) = 0.54***; fear of blood, injury (FSS) = 0.32** pain impact (PCL-e) = 0.27**; pain intensity (MPQ) = 0.21** TSK_harm_: catastrophising (PCS) = 0.47***; fear of blood, injury (FSS) = 0.32**; pain impact (PCL-e) = 0.23**; pain intensity (MPQ) = 0.24** TSK_fear_: catastrophising (PCS) = 0.52***, fear of blood, injury (FSS) = 0.37**; pain impact (PCL-e) = 0.38***; pain intensity (MPQ) = 0.25** TSK_Avoidance of activity_: catastrophising (PCS) = 0.35*** **Regression: Prediction** Dependent: Disability: *β* TSK_total_ = 0.44* (d) —	(a) — (b) —

*Dutch 13 items* Goubert et al. [51]	(a) 277 CLBP+FMS (b) 33.6% (c) 41.33 (d) 97.9 + 116.4	(a) ***α*** (1) TSK_total_ CLBP = 0.80; FMS = 0.82 (2) TSK_activity avoidance _CLBP = 0.73; FMS = 0.76 (3) TSK_pathologic somatic focus_ CLBP = 0.70; FMS = 0.70 (b) —	(a) — (b) **Construct validity: CFA:** *2 factors:* activity avoidance; pathological somatic focus (c) —	(a) — (b) —

*Dutch 11 items* Roelofs et al. [52]	(a) 1109 UED + 2825 CP (b) 33% + no info (c) 41.7 + no info (d) no info	(a) ***α*** *UED * TSK_11_ = 0.77 Chronic pain TSK_11_ = 0.75–0.80 (b) —	(a) — (b) **Construct validity:** CFA: *2 factors* (11 items): Somatic focus, Activity avoidance (c) **Criterion related: Concurrent** TSK_SF_ pain related: functioning (DASH) = 0.23**; pain intensity = 0.24** physical: sports (DASH) = 0.18**; work (DASH) = 0.31** TSK_AA_ pain related: functioning (DASH) = 0.32**; pain duration = 0.14**; pain intensity = 0.19** *physical:* sports (DASH) = 0.25**; work (DASH) = 0.27** **Regression: Predictive** (1) Dependent: Disability: *β* TSK_AA _= 0.13*** (2) Dependent: Disability, work: *β* TSK_SF_ = 0.16***: *β* TSK_AA _= 0.06* (3) Dependent: Disability, sports: *β* TSK_AA_ = 0.18*** (d) —	(a) — (b) —

*English 4 items* Burwinkle et al. [53]	(a) 233 FMS (b) 0% (c) 43.79 (d) 122.4	(a) ***α*** TSK_4_ = 0.71 (b) —	(a) — (b) Construct validity: PCA: 1 factor (54.2%) (c) —	(a) — (b) —

*English 11 items* Woby et al. [54]	(a) 111 + 104 CLBP (b) no info (c) 43.4 + 44.4 (d) 79.2+80.4	(a) ***α*** TSK_total_ = 0.76 TSK_11_ = 0.79 (b) **ICC** TSK = 0.82 (c) SEM = 3.16 item-total <.20 (4, 8, 9, 12, 14, 16)	(a) — (b) **Construct validity:** Correlations between change scores TSK_total_: Disability (RDQ) = 0.50***; Pain (VAS) = 0.22* TSK_11_: Disability (RDQ) = 0.51***; Pain (VAS) = 0.27** (c) **Criterion related: Predictive** Regression Dependent: change in disability; *β* TSK_total_ = 0.39; *β* TSK_11_ = 0.38 (d) —	(a) **SRM** TSK_total_ = −1.19; TSK_11_ = −1.11 (b) **AUC** TSK_total_ = 0.70; TSK_11_ = 0.71 (c) **Sensitivity** TSK_total_ = 71%; TSK_11_ = 66% **Specificity** TSK_total_ = 63%; TSK_11_ = 67% TSK_total_ = 4 pts; TSK_11_ = 4 pts

*English 13 items* Clark et al. [55]; French et al. [56]	(a) 167 veterans; 200 CBP + NP (b) 100%; 46% (c) 47.6; 40.0 (d) 180; 16.7	(a) ***α*** TSK_total_ = 0.86; 0.84 TSK_taa_ = 0.78 TSK_tsf_ = 0.70 (b) — (c) **Item-total** = 0.29–0.69	(a) — (b) **Construct validity (EFA):** *2 factors*: activity avoidance, pathological somatic focus *3 factors * (c) **Criterion-related validity: concurrent** *pain related:* disability (Quebec) = 0.30**; pain interference (MVAS) = 0.39**; pain intensity (VAS) = 0.23** *Psychosocial:* anxiety (STAI) = 0.28**; catastrophising (PCS) = 0.53**; depression (BDI) = 0.34**; FABQ_pa_ = 0.35**; FABQ_work_ = 0.34** **Regression** (1) Dependent: Pain interference (MVAS); *β* (TSK) = 0.29*** (2) Dependent: Disability (Quebec); *β* (TSK) = 0.22*** (d) —	(a) — (b) —

*Norwegian* Damsgård et al. [57]	(a)120 patients: 48 LBP, 72 WP (b)LBP: 42% women, 58% men WP: 58% women, 42% men (c)mean age 42 (SD 10) (d) —	(a) — (b) Reproducibility: Pearson separation reliability = 0.87	(a) — (b) Mean item fit = 0.26 (SD 0.86); Mean person fit = −0.17 (SD 1.15) Targeting: Item distribution −3.2 to 3.2 Pearsons location mean = −0.21 (SD 1.12) Invariance: Item 10 varied for gender Analysis of residual pattern: Unidimensional underlying construct according to binomial test CI for probability 0.05 to 0.13 (c) —	(a) — (b) —

*Swedish 17 items* Lundberg et al. [7]; Bunketorp et al. [58]	(a) 102 CLBP; 46 WAD (b) 49% (c) 45.3 (d) 66; 1.5–3	(a) ***α***TSK = 0.81 (b) **ICC** = 0.91 r_p_ = 0.91*** RP = −0.01, CI = (−0.12; 0.14) RC = −0.08, CI = (−0.25; 0.09) RV = 0.21, CI = (0.04; 0.38) Kappa = 0.0 (c) **Item-total** <.40	(a) **Content validity:** Pilot study, Translation, back translation (b) **Construct validity:** PCA: *5 factors* (59%) + known group technique (c) —	(a) — (b) —

Abbreviations in study population: CBP: Chronic back pain, CLBP: Chronic Low Back Pain, CP: Chronic Pain, FMS: Fibromyalgia Syndrome, LBP: Low Back Pain, NP: Neck pain, RA: Rheumatoid Arthritis, UED: Upper Extremity Disorder, WAD: Whiplash Associated Disorder.

Abbreviations in reliability: *α*: Cronbach's alpha, ICC: Intraclass Correlation Coefficient, rp: Pearson's product moment correlation coefficient.

Abbreviations in validity: AIMS: Arthritis Impact Measurement Scales, ASE: Arthritis Self-Efficacy scale, ASI: Anxiety Sensitivity Index, BDI: Beck Depression Inventory, CCE: Carrying Capacity Evaluation; CFA: Confirmatory Factor Analysis, CSAQ: Cognitive Somatic Anxiety Questionnaire, CSQ: Coping Strategy Questionnaire, DASH: Disabilities of the Arm, Shoulder and Hand questionnaire, EFA: Exploratory Factor Analysis, FCE: Functional Capacity Evaluation, FFbH-R: physical functioning scale (Funktionsfragebogen Hannover-Rücken), FLP: Functional Limitations Profile, FSS: Fear Survey Schedule, GAS: General Activity Scale, HADS: Hospital Anxiety Depression Scale, HSCL: Hopkins Symptom Check list, McGill: Mc Gill pain questionnaire, MPI: Multidimensional Pain Inventory, MPQ: Mc Gill pain questionnaire, MSPQ: Modified Somatic Perceptions Questionnaire, NEM: Negative Emotionality Questionnaire, NPQ: Neck Pain Questionnaire, NRS: Numerical Rating Scale, ODQ: Oswestry Disability Questionnaire, PBC: Pain Behaviour Checklist, PCA: Principal Component Analysis, PCL: Pain Cognition List, PCS: Pain Catastrophising Scale, PDI: Pain Disability Index, PRSS-CAT: Pain-Related Self-Statements Scale, Quebec score: The Quebec Back Pain Disability Scale, PSS: Pain Severity Scale, PVAQ: Pain Vigilance and Awareness Questionnaire, RDQ; RM: Roland Morris Disability Questionnaire, SF36: Short Form36, STAI: Spielberger State and Trait Anxiety Inventory, VAS: Visual Analogue Scale, ZDI: Zung Depression Inventory.

Abbreviations in responsiveness: AUC: Area Under Curve, SRM: Standardised Response Mean.
